# A U-shaped association between the triglyceride to high-density lipoprotein cholesterol ratio and the risk of incident type 2 diabetes mellitus in Japanese men with normal glycemic levels: a population-based longitudinal cohort study

**DOI:** 10.3389/fendo.2023.1180910

**Published:** 2023-09-21

**Authors:** Bei Song, Kun Wang, Weilin Lu, Xiaofang Zhao, Tianci Yao, Ting Liu, Guangyu Gao, Haohui Fan, Chengyun Liu

**Affiliations:** Department of Geriatrics, Union Hospital, Tongji Medical College, Huazhong University of Science and Technology, Wuhan, China

**Keywords:** triglyceride to high-density lipoprotein cholesterol ratio, type 2 diabetes mellitus, cohort study, normal glycemic level, Japanese men

## Abstract

**Background:**

Several studies have verified that a high baseline TG/HDL-C ratio is a risk factor for incident type 2 diabetes mellitus (T2DM). However, for low baseline TG/HDL-C levels, the findings were inconsistent with ours. In addition, the association between baseline TG/HDL-C ratio and the risk of incident T2DM in Japanese men with normal glycemic levels is unclear. As a result, our study further investigated the relationship between baseline TG/HDL-C and the risk of incident T2DM in Japanese men with normal glycemic levels.

**Methods:**

This was a secondary longitudinal cohort study. We selected 7,684 male participants between 2004 and 2015 from the NAGALA database. A standardized Cox regression model and two piecewise Cox regression models were used to explore the relationship between the baseline high-density lipoprotein cholesterol ratio (TG/HDL-C) and incident T2DM.

**Results:**

During a median follow-up of 2,282 days, 162 men developed incident T2DM. In the adjusted model, the baseline TG/HDL-C ratio was strongly associated with the risk of incident T2DM, and no dose-dependent positive association was observed between the baseline TG/HDL-C ratio and incidence of T2DM throughout the baseline TG/HDL-C quartiles. Two-piecewise linear regression analysis showed a U-shaped association between baseline TG/HDL-C ratio and incidence of incident T2DM. A baseline TG/HDL-C ratio below 1.188 was negatively associated with incident T2DM (H.R. = 0.105, 95% CI = 0.025, 0.451; P = 0.002). In contrast, a baseline TG/HDL-C ratio >1.188 was positively associated with incident T2DM (H.R. = 1.248, 95% CI = 1.113, 1.399; P<0.001). The best TG/HDL-C threshold for predicting incident T2DM was 1.8115 (area under the curve, 0.6837).

**Conclusion:**

A U-shaped relationship between baseline TG/HDL-C ratio and incident T2DM in Japanese men with normal glycemic levels was found.

## 1 Introduction

Diabetes is one of the most common chronic metabolic diseases (1). Diabetes has been identified by the International Diabetes Federation (IDF) as one of the fastest-growing global health emergencies of the 21st century with a profound economic impact. In 2021, there were approximately 537 million people with diabetes worldwide, and approximately 643 million people will have diabetes by 2030, and 783 million by 2045. The number of people with diabetes is increasing in Japan. In 2021, Japanese adults had the world’s fifth highest health spending on diabetes, at approximately 35.6 billion dollars. Type 2 diabetes mellitus (T2DM), is the most common form of diabetes, accounting for 90% of all cases (2). Therefore, the prevention and early diagnosis of type 2 diabetes are crucial.

In the pathogenesis of T2DM, insulin resistance (IR) plays an important role (3). Lipid metabolism disorders are the main cause of IR pathophysiology (4). High levels of triglycerides (TG) and low levels of high-density lipoprotein cholesterol (HDL-C) are closely associated with IR and T2DM (5). TG/HDL-C is closely related to IR according to previous studies, which has been advocated as a simple clinical indicator of IR (6, 7). Three studies based on Chinese populations and one study based on Singapore Chinese men and women consistently found that triglyceride to high-density lipoprotein cholesterol (TG/HDL-C) ratio was positively associated with the risk of T2DM (8–11). A dose-dependent relationship between TG/HDL-C ratio and the risk of incident T2DM was also found in a study of Korean adults (12). However, one study in Iran showed that TG/HDL-C ratio was not associated with the risk of incident T2DM (13). However, in a Chinese population-based study, a stronger association between TG/HDL-C and incident diabetes mellitus was found in people with baseline fasting plasma glucose (FPG) of less than 6.1 mmol/L, which indicates that our study should further investigate the relationship between TG/HDL-C and incident T2DM in individuals with FPG less than 6.1 mmol/L (14). We only included participants with fasting blood glucose less than 6.1 mmol/L and HbA1c less than 5.7% at baseline to exclude people with prediabetes at baseline. The most important finding was that our study found a specific U-shaped relationship between TG/HDL-C and the risk of incident T2DM in Japanese men which was completely different from previous studies in other countries.

Our study was a population-based cohort study to examine the association between TG/HDL-C ratio and the risk of incident T2DM in Japanese men aged 18–69 years. Women were excluded because we found an interesting U-shaped relationship between TG/HDL-C and the risk of incident T2DM among Japanese men but not among Japanese women. We hope that our study will contribute to the diagnosis and prevention of incident T2DM in Japanese men and provide a basis for future clinical and mechanistic studies.

## 2 Methods and materials

### 2.1 Participants

In this study, we used data from the NAGALA (NAFLD in the Gifu Area, Japan, Longitudinal Analysis) database to conduct a secondary analysis. Between 2004 and 2015, these data were obtained from the Murakami Memorial Hospital Examination Project. A total of 12,498 men and 8,446 women were enrolled in this study. However, because our analysis found a unique threshold effect between TG/HDL-C ratio and incident T2DM in Japanese men with normal glycemic levels, we included only 12,498 men. Furthermore, we excluded participants who had liver disease (e.g., alcoholic fatty liver disease and viral hepatitis), any medication usage, and excessive drinking habits at baseline. Participants who lacked covariates such as height, TG/HDL-C ratio, exercise habits, alcohol consumption, or abdominal ultrasonography were also excluded. Finally, we only included participants with fasting blood glucose less than 6.1 mmol/L and HbA1c less than 5.7% at baseline, because we wanted to exclude people with prediabetes at baseline. The study was approved by the Murakami Memorial Hospital Ethics Committee, and the participants signed written informed consent forms.

### 2.2 Data collection and measurements

Data were collected using a self-report questionnaire that included information about the participants’ lifestyle (alcohol and smoking habits and physical activity) and history of drug use. Alcohol consumption, defined as the average weekly alcohol intake, was estimated by asking participants about their average weekly alcohol intake in the month prior to the examination. To facilitate statistical analysis, four groups were formed as follows: non-drinker,<40 g/week; light drinker, 40 g/week–140 g/week; moderate drinker, 140 g/week–280 g/week; and severe drinker, >280 g/week. Three groups were formed based on smoking status: never smokers (never smoked), former smokers (previously smoked but quit before the baseline examination), and current smokers (during the baseline examination, he smoked). Exercise habits were defined as participation in exercise once a week or more regularly. In the original data, fatty liver was diagnosed mainly using abdominal ultrasonography. The gastroenterologist diagnosed the participants with fatty liver based on liver brightness and contrast. Hepatitis B antigen and hepatitis C antibody-positive patients were defined as those with viral hepatitis. T2DM was described as HbA1c greater than or equal to 6.5%and fasting blood glucose greater than or equal to 7 mmol/L after physical examination at follow-up or as self-reported by the participants. However, because the oral glucose tolerance test (OGTT) was not performed during diagnosis, the incidence rate of T2DM may be underestimated.

### 2.3 Definition of TG/HDL-C


TG/HDL−C=triglyceride (mmol/L)/high−density lipoprotein cholesterol(mmol/L)

### 2.4 Statistical analysis

**Figure 1:**
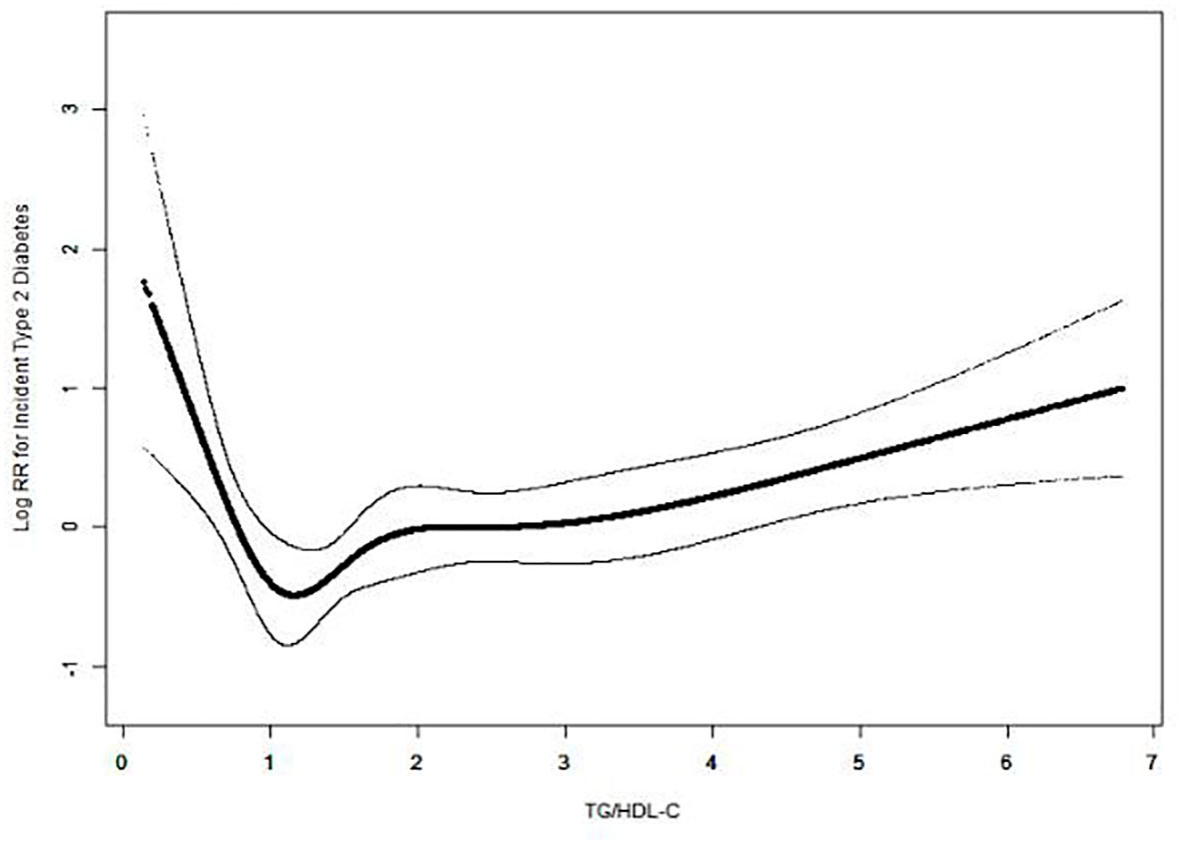
U-shaped association between TG/HDL-C ratio and incident T2DM. A nonlinear association between TG/HDL-C ratio and incident T2DM was observed in a generalized additive model (GAM). The solid black line represents the smooth curve fit between the TG/HDL-C ratio and the incidence of diabetes. Dotted curves represent 95% CI of the fit. All were adjusted for age, fatty liver, body weight, waist circumference, BMI, total cholesterol, ALT, AST, GGT, systolic blood pressure, diastolic blood pressure, alcohol consumption, smoking status, and exercise habits.

**Figure 2:**
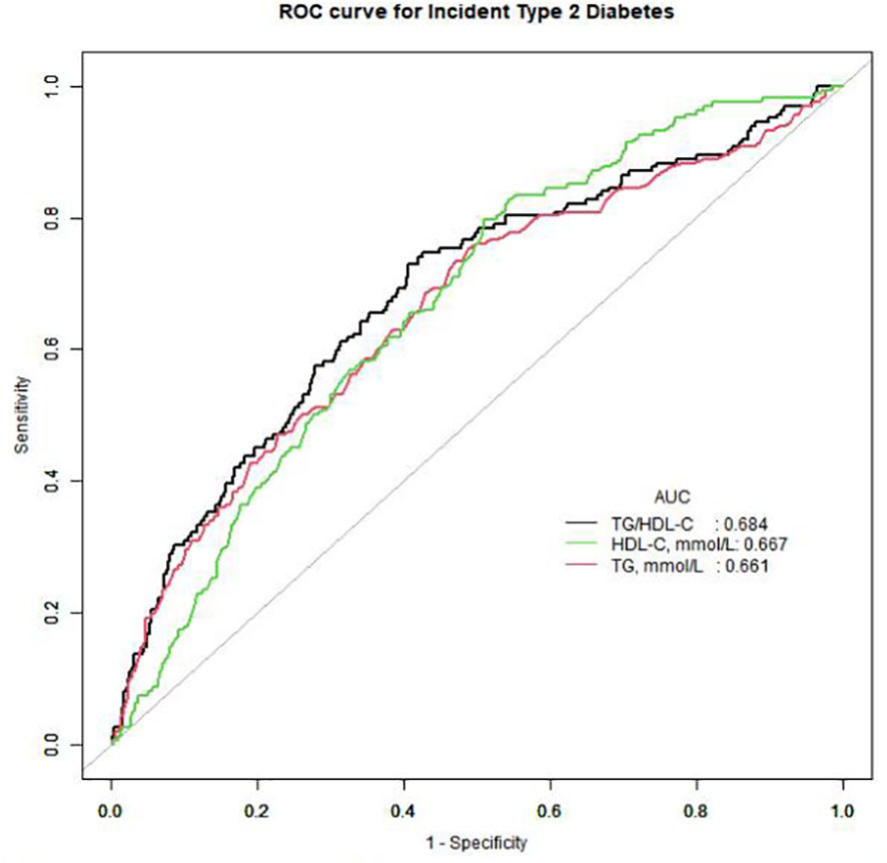
Receiver operating characteristic (ROC) curve analyses to predict incident T2DM. AUC, area under the curve; TG, triglyceride; HDL-cholesterol, high-density lipoprotein-cholesterol; TG/HDL-C, triglyceride to high-density lipoprotein cholesterol ratio.

All statistical analyses in our study used the statistical packages R and EmpowerStats (15). As shown in **Table 1**, the TG/HDL-C ratio was divided into four groups (Q1–Q4). To make the analysis results more reliable and accurate, we divided the participants into four equal groups based on the TG/HDL-C ratio. Continuous variables were expressed as mean ± standard deviation (SD). The Kolmogorov–Smirnov test was used to assess the normality of the data, and the Student’s t-test was used to assess statistical differences between groups for continuously normally distributed variables. If the normality assumption was not met, the Mann–Whitney U test was used for statistical analysis. Categorical data were expressed as frequencies (percentages). Statistical differences between categorical variables were analyzed using the chi-squared test. We performed Cox regression analysis to assess the independent effect of the baseline TG/HDL-C ratio on the incidence of T2DM (**Table 2**). We used four models: (1) a crude model without adjustment. (2) Model I adjusted for age and BMI. (3) Model II adjusted for age, BMI, fatty liver, waist circumference, body weight, alcohol consumption, smoking status, exercise habits, systolic blood pressure, and diastolic blood pressure. (4) Model III was adjusted for all variables with P<0.001 in the univariate analysis (baseline age, BMI, fatty liver, waist circumference, body weight, alcohol consumption, smoking status, exercise habits, systolic blood pressure, diastolic blood pressure, total cholesterol, HbA1c, fasting plasma glucose, GGT, ALT, and AST) (**Supplementary Table 1**). Finally, in **Table 3**, we used a two-piecewise linear regression model to examine the threshold effect of the baseline TG/HDL-C ratio on incident T2DM, which was in terms of the smoothing plot (**Figure 1**). We used the maximum likelihood model to further calculate the inflection points. In **Figure 2** and **Table 4**, receiver operating characteristic (ROC) curve analysis was used to calculate the area under the curve (AUC) and the best threshold, which showed the predictive value of the TG/HDL-C, TG, and HDL-C for incident T2DM risk.

**Table 1:** Baseline variables according to the quartile of TG/HDL-C.

Variable	TG/HDL-C				*P*-value
	Quartile 1	Quartile 2	Quartile 3	Quartile 4	
	(0.1448–0.9978)	(1.0000–1.5957)	(1.5960–2.6075)	(2.6078–6.7895)	
**N**	**1,900**	**1,942**	**1,921**	**1,921**	
**Case of Incident T2DM**	**19 (1.00%)**	**18 (0.93%)**	**45 (2.34%)**	**80 (4.16%)**	**<0.001**
**Age, yr**	**42.41 ± 9.10**	**43.83 ± 9.11**	**44.23 ± 8.83**	**44.71 ± 8.40**	**<0.001**
**Body Weight, kg**	**62.97 ± 8.27**	**65.24 ± 8.83**	**68.04 ± 9.88**	**71.34 ± 9.82**	**<0.001**
**Waist circumference, cm**	**75.69 ± 6.70**	**78.66 ± 6.94**	**81.49 ± 7.53**	**84.40 ± 7.19**	**<0.001**
**BMI, kg/m^2^**	**21.45 ± 2.43**	**22.35 ± 2.59**	**23.30 ± 2.89**	**24.44 ± 2.82**	**<0.001**
**HDL-C, mmol/L**	**1.65 ± 0.36**	**1.38 ± 0.25**	**1.22 ± 0.21**	**1.06 ± 0.19**	**<0.001**
**TG, mmol/L**	**0.49 ± 0.14**	**0.76 ± 0.16**	**1.08 ± 0.22**	**1.76 ± 0.47**	**<0.001**
**Total cholesterol, mmol/L**	**4.86 ± 0.79**	**5.00 ± 0.79**	**5.20 ± 0.81**	**5.45 ± 0.86**	**<0.001**
**ALT, IU/L**	**19.13 ± 9.83**	**21.20 ± 10.69**	**24.20 ± 13.21**	**28.75 ± 16.55**	**<0.001**
**AST, IU/L**	**18.73 ± 7.88**	**18.63 ± 6.52**	**19.29 ± 6.95**	**21.05 ± 8.62**	**<0.001**
**GGT, IU/L**	**20.07 ± 15.10**	**22.80 ± 16.82**	**26.08 ± 21.67**	**31.15 ± 24.63**	**<0.001**
**HbA1c, %**	**5.14 ± 0.30**	**5.15 ± 0.31**	**5.18 ± 0.33**	**5.21 ± 0.34**	**<0.001**
**Fasting plasma glucose, mmol/L**	**5.21 ± 0.37**	**5.26 ± 0.35**	**5.30 ± 0.35**	**5.36 ± 0.36**	**<0.001**
**Systolic blood pressure, mmHg**	**115.16 ± 13.06**	**117.65 ± 13.51**	**118.95 ± 14.12**	**121.87 ± 14.45**	**<0.001**
**Diastolic blood pressure, mmHg**	**71.90 ± 9.61**	**74.06 ± 9.36**	**75.03 ± 9.78**	**77.30 ± 10.01**	**<0.001**
**Follow-up duration, days**	**2,041.83 ± 1,358.25**	**2,307.72 ± 1,422.34**	**2,348.04 ± 1,413.71**	**2,425.88 ± 1,421.53**	**<0.001**
**Fatty liver**					**<0.001**
**No**	**1,770 (93.16%)**	**1,646 (84.76%)**	**1,390 (72.36%)**	**1,033 (53.77%)**	
**Yes**	**130 (6.84%)**	**296 (15.24%)**	**531 (27.64%)**	**888 (46.23%)**	
**Habit of exercise**					**<0.001**
**No**	**1,427 (75.11%)**	**1,551 (79.87%)**	**1,577 (82.09%)**	**1,641 (85.42%)**	
**Yes**	**473 (24.89%)**	**391 (20.13%)**	**344 (17.91%)**	**280 (14.58%)**	
**Alcohol consumption**					**0.037**
**Never**	**1,166 (61.37%)**	**1,194 (61.48%)**	**1,228 (63.93%)**	**1,259 (65.54%)**	
**Light**	**329 (17.32%)**	**348 (17.92%)**	**306 (15.93%)**	**276 (14.37%)**	
**Moderate**	**287 (15.11%)**	**270 (13.90%)**	**273 (14.21%)**	**250 (13.01%)**	
**Severe**	**118 (6.21%)**	**130 (6.69%)**	**114 (5.93%)**	**136 (7.08%)**	
**Smoking status**					**<0.001**
**Never**	**815 (42.89%)**	**678 (34.91%)**	**604 (31.44%)**	**598 (31.13%)**	
**Past**	**573 (30.16%)**	**612 (31.51%)**	**566 (29.46%)**	**529 (27.54%)**	
**Current**	**512 (26.95%)**	**652 (33.57%)**	**751 (39.09%)**	**794 (41.33%)**	

Continuous variables are presented as mean ± S.D. or as median (Q1–Q4). Categorical data are presented as frequencies (percentages).

Q, quartile; ALT, alanine aminotransferase; AST, aspartate aminotransferase; GGT, gamma-glutamyl transpeptidase; DBP, diastolic blood pressure; SBP, systolic blood pressure; HDL-C, high-density lipoprotein-cholesterol; TG, triglyceride; TG/HDL-C, triglyceride to high-density lipoprotein cholesterol ratio; HbA1c, Hemoglobin A1c; BMI, body mass index.

**Table 2:** Associations of baseline TG/HDL-C with incident T2DM.

	Incident T2DM			
	Crude Model	Model I	Model II	Model III
	H.R. (95%CI) *P-*value	H.R. (95%CI) *P-*value	H.R. (95%CI) *P-*value	H.R. (95%CI) *P-*value
**TG/HDL-C continuous**	**1.469 (1.340, 1.610)<0.001**	**1.301 (1.175, 1.440)<0.001**	**1.196 (1.073, 1.332) 0.001**	**1.188 (1.063, 1.327) 0.002**
**TG/HDL-C quartile**				
**Q1**	**1.000**	**1.000**	**1.000**	**1.000**
**Q2**	**0.755 (0.396, 1.439) 0.393**	**0.597 (0.313, 1.141) 0.118**	**0.490 (0.255, 0.941) 0.032**	**0.486 (0.253, 0.936) 0.031**
**Q3**	**1.872 (1.095, 3.201) 0.022**	**1.214 (0.701, 2.102) 0.488**	**0.910 (0.520, 1.593) 0.741**	**0.891 (0.505, 1.573) 0.692**
**Q4**	**3.151 (1.910, 5.199)<0.001**	**1.619 (0.961, 2.727) 0.070**	**1.050 (0.609, 1.810) 0.861**	**1.012 (0.578, 1.773) 0.967**

Crude model adjusted for None.

Model I adjusted for baseline age and BMI.

Model II adjusted for baseline age, BMI, fatty liver, waist circumference, body weight, exercise habits, alcohol consumption, smoking status, systolic blood pressure, and diastolic blood pressure.

Model III adjusted for baseline age, fatty liver, BMI, waist circumference, body weight, exercise habits, alcohol consumption, smoking status, systolic blood pressure, diastolic blood pressure, total cholesterol, GGT, ALT, and AST.

HR, hazard ratio; CI, confidence intervals; Q, quartile; ALT, alanine aminotransferase; AST, aspartate aminotransferase; GGT, gamma-glutamyl transpeptidase; DBP, diastolic blood pressure; SBP, systolic blood pressure; HDL-cholesterol, high-density lipoprotein-cholesterol; TG/HDL-C, triglyceride to high-density lipoprotein cholesterol ratio; BMI, body mass index.

**Table 3:** Threshold effect analysis of baseline TG/HDL-C ratio and incident T2DM using piece-wise linear regression.

	Incident T2DM					
	Crude model		Model I		Model II	
	HR (95%CI)	*P*-value	H.R. (95%CI)	*P-*value	H.R. (95%CI)	*P*-value
**TG/HDL-C<1.188**	**0.791 (0.294, 2.126)**	**0.642**	**0.280 (0.101, 0.778)**	**0.015**	**0.105 (0.025, 0.451)**	**0.002**
**TG/HDL-C >1.188**	**1.514 (1.365, 1.679)**	**<0.001**	**1.297 (1.158, 1.454)**	**<0.001**	**1.248 (1.113, 1.399)**	**<0.001**
**Likelihood ratio test *p***		**0.235**		**0.009**		**0.003**

Crude model adjusted for None.

Model I was adjusted for baseline age, fatty liver, BMI, waist circumference, and body weight.

Model II was adjusted for baseline age, fatty liver, BMI, waist circumference, ALT, AST, body weight, exercise habits, GGT, total cholesterol, alcohol consumption, smoking status, systolic blood pressure, and diastolic blood pressure.

HR, hazard ratio; CI, confidence interval; TG/HDL-C, triglyceride to high-density lipoprotein cholesterol ratio.

**Table 4:** AUC with the 95% CI of TG/HDL-C, HDL-C and TG for predicting incident T2DM.

Test	ROC area (AUC)	95%CI low	95%CI up	Best threshold	Specificity	Sensitivity
**TG/HDL-C**	**0.6837**	**0.6397**	**0.7277**	**1.8115**	**0.575**	**0.7469**
**TG**	**0.6611**	**0.6158**	**0.7064**	**0.8976**	**0.511**	**0.7531**
**HDL-C**	**0.6674**	**0.6298**	**0.705**	**1.2891**	**0.49**	**0.7963**

AUC, area under the curve; CI, confidence interval; TG, triglyceride; HDL-cholesterol, high-density lipoprotein-cholesterol; TG/HDL-C, triglyceride to high-density lipoprotein cholesterol ratio.

## 3 Results

### 3.1 Study population description based on TG/HDL-C quartiles

**Figure 3:**
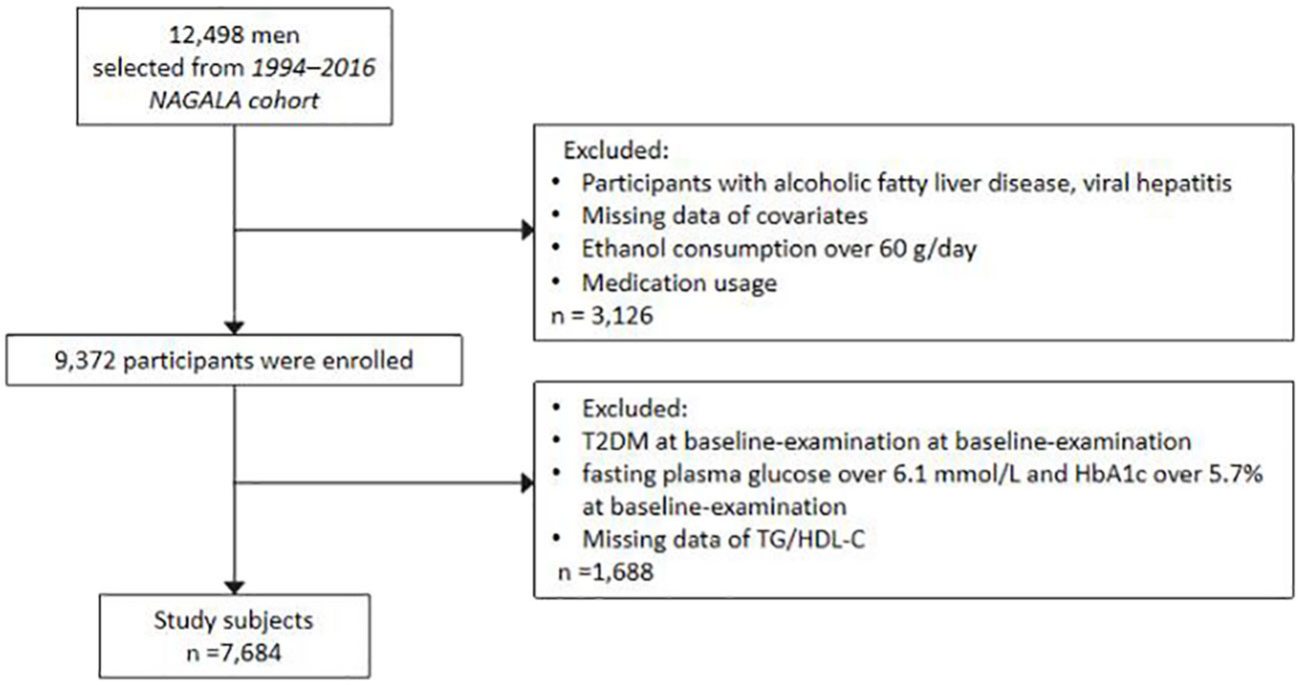
Flow chart showing the exclusion criteria of participants. NAGALA, NAfld in Gifu Area, Longitudinal Analysis; T2DM, type 2 diabetes mellitus; TG/HDL-C, triglyceride to high-density lipoprotein cholesterol ratio.

A total of 12,498 men and 8,446 women were initially enrolled in the NAGALA cohort study. However, because our analysis found a unique threshold effect between incident T2DM and TG/HDL-C ratio in Japanese men with normal glycemic levels, we included only 12,498 men. Furthermore, 788 men were excluded due to a lack of covariates, such as height, TG/HDL-C, exercise habits, alcohol consumption, or abdominal ultrasonography. The 2,622 men who had liver disease, medication usage, and excessive drinking habits at baseline were also excluded. A total of 677 men were also excluded, including 265 with type 2 diabetes at baseline and 667 with fasting blood glucose of more than 6.1 mmol/L or HbA1c >5.7% at baseline. Therefore, 7,684 men were included in this cohort study (**Figure 3**).

The baseline characteristics of the study participants are shown in **Table 1**. In our cohort, the mean age was 43.80 ± 8.90 years. At a mean follow-up of 2,281.59 ± 1,411.46 days, 162 (2.11%) subjects developed incident T2DM. All baseline variables showed statistically significant differences between the quartiles. Participants in the higher TG/HDL-C quartiles were more likely to be incident T2DM patients, older, fatty liver patients, current smokers, non-drinkers, severe drinkers, persons who had no exercise habits, and had higher body weight, BMI, triglycerides, total cholesterol, fasting plasma glucose, ALT, AST, GGT, HbA1c, systolic blood pressure, diastolic blood pressure, larger waist circumference, but less likely to be light drinkers, moderate drinkers, non-smokers, past smokers, and those with lower HDL-cholesterol levels (P<0.001).

### 3.2 The association between incident T2DM and baseline TG/HDL-C

We performed Cox regression analysis to assess the independent effect of the baseline TG/HDL-C ratio on the incidence of T2DM (**Table 2**). In the crude model, the risk of T2DM was prominently associated with baseline TG/HDL-C in men (P<0.001). In Model I, the association remained significant after adjusting for age and BMI. In addition, Model II was further adjusted for waist circumference, body weight, fatty liver, alcohol consumption, smoking status, systolic blood pressure, diastolic blood pressure, and exercise habits, which did not alter the significant association among men (P<0.001), but it was weaker than Model I. Model III adjusted for all variables (baseline age, waist circumference, body weight, fatty liver, BMI, alcohol consumption, exercise habits, smoking status, diastolic blood pressure, systolic blood pressure, total cholesterol, AST, ALT, and GGT) associated with incident T2DM in univariate analysis, and significant correlations remained (P<0.05) (**Supplemental Table 1**). We then divided the participants into four groups based on the baseline TG/HDL-C ratio. Neither model showed a dose-dependent positive relationship between the baseline TG/HDL-C quartile and the risk of T2DM. In Model III Compared to the TG/HDL-C quartile 1, the risk of incident T2DM did not increase significantly in quartiles 2 (HR = 0.443, 95% CI = 0.229, 0.856; P = 0.015), 3 (HR = 0.841, 95% CI = 0.478, 1.479; P = 0.548), and 4 (HR = 0. 0.848, 95% CI = 0.487, 1.478; P = 0.561) of TG/HDL-C. These findings illustrate the significant nonlinear relationship between baseline TG/HDL-C ratio and incident T2DM.

### 3.3 Two piecewise linear regression analysis and threshold effect analysis of TG/HDL-C on the Incident T2DM

Because previous multiple regression analyses indicated a nonlinear association between baseline TG/HDL-C ratio and the risk of incident T2DM, a threshold effect analysis with a smooth function was used to further clarify the association. Interestingly, smooth curves adjusted for multiple confounders showed a U-shaped association between TG/HDL-C ratio and the risk of T2DM in Japanese men (**Figure 1**). According to the two piecewise linear regression models, after adjusting for confounding variables, the baseline TG/HDL-C ratio was negatively correlated with the log-relative risk of incident T2DM when the baseline TG/HDL-C ratio was less than 1.188. After adjusting for baseline age, waist circumference, body weight, fatty liver, BMI, alcohol consumption, exercise habits, smoking status, diastolic blood pressure, systolic blood pressure, total cholesterol, AST, ALT, and GGT when TG/HDL-C ratio was less than 1.188, the risk of incident T2DM in Japanese men decreased by nearly 89.5% for each unit increase in TG/HDL-C ratio (HR = 0.105, 95% CI = 0.025, 0.451; P = 0.002). In contrast, a baseline TG/HDL-C ratio >1.188 was significantly positively associated with the risk of T2DM (HR = 1.248, 95% CI = 1.113, 1.399; P<0.001) (**Table 3**).

### 3.4 Predictive value of TG/HDL-C in incident T2DM

To compare the predictive value of TG/HDL-C with that of TG and HDL-C, an ROC curve was drawn, and the area under the curve (AUC) was calculated. The area under the curve (AUC) of TG/HDL-C was 0.6837 (0.6397, 0.7277), which was larger than that of TG and HDL-C (**Figure 2**). The best threshold, specificity, and sensitivity of TG/HDL-C ratio were 1.8115, 0.575, and 0.7469, respectively (**Table 4**).

## 4 Discussion

### 4.1 Comparisons with other studies and what does the current work add to the existing knowledge

To our knowledge, our study is the first to describe a U-shaped association between baseline TG/HDL-C ratio and the risk of incident T2DM in Japanese men with normal glycemic levels. In addition, we identified a turning point (TG/HDL-C ratio = 1.188) using threshold effect analysis and a two-piecewise linear regression model. According to the two-piecewise linear regression model, when the TG/HDL-C ratio was greater than 1.188, the risk of T2DM increased significantly with an increase in baseline TG/HDL-C ratio, which was consistent with previous studies in other countries. Cheng et al. found that the incidence of T2DM increased with an increase in TG/HDL-C ratio in rural China (9). Another retrospective cohort study based on a Chinese population showed that participants with TG/HDL-C in quartiles 2, 3, and 4 had a higher risk of developing T2DM than those in quartile 1 (8). However, Incident T2DM decreased in quartile 2 compared to quartile 1, which verified that there was a nonlinear relationship (U-shaped) between TG/HDL-C ratio and incident T2DM. At the same time, the results supported that when TG/HDL-C ratio was less than 1.188, it had a protective effect on new-onset type 2 diabetes in Japanese men.

This was a population-based cohort study to examine the association between TG/HDL-C ratio and the risk of incident T2DM in Japanese men aged 18-69–years. Interestingly, we found that a lower baseline TG/HDL-C ratio (TG/HDL-C<1.188) significantly altered the association between TG/HDL ratio and incident T2DM risk. After adjusting for confounding factors such as baseline age, fatty liver, BMI, waist circumference, ALT, AST, body weight, exercise habits, GGT, total cholesterol, HbA1c, alcohol consumption, smoking status, fasting plasma glucose, systolic blood pressure, and diastolic blood pressure, for each unit increase in baseline TG/HDL-C below the threshold, the risk of developing incident T2DM in Japanese men was decreased by nearly 89.5%. This result was inconsistent with those of studies conducted in China, South Korea, Singaporean Chinese, and Iran. Among them, TG/HDL-C ratio was not associated with diabetes incidence in the Iranian population (13). In addition, studies in other countries reported an increased risk of diabetes or T2DM in all TG/HDL-C groups (8–12, 14). However, most studies in these countries only excluded participants with baseline FPG greater than 7 mmol/L and did not exclude people with prediabetes (HbA1c greater than 5.7% and FPG greater than 6.1 mmol/L) (9, 12, 14). Some studies did not specify the specific criteria for the inclusion of the population or diagnosis of T2DM (8). In addition, the TG/HDL-C range in our study was wider than that in these three studies, which may partly account for the divergent results, possibly because some participants with prediabetes were included in these studies. In three studies on the Chinese population, a nonlinear relationship was found after adjusting for confounding factors (8, 9, 14). The risk of T2DM increased significantly only in quartile 4 of TG/HDL-C, but not in quartiles 2 and 3, compared with quartile 1. In addition, a study of Singapore Chinese and Korean adults did not conduct bilinear regression and smooth function analysis, and they were grouped into three groups, so specific trends could not be observed (10, 12). In conclusion, a U-shaped association between baseline TG/HDL-C ratio and the development of incident T2DM was found in Japanese men with normoglycemic levels, possibly due to different regions, different population screening patterns, and a broader range of baseline TG/HDL-C ratio.

This study has several important clinical implications. First, the association between higher TG/HDL-C ratio and the risk of incident T2DM may be due to insulin resistance. The specific mechanism remains unclear, but some studies have shown that dyslipidemia is a vital pathogenesis of insulin resistance (4). Lipotoxicity, endoplasmic reticulum (ER) stress, and inflammation are the widely accepted mechanisms for inducing IR (3, 16). Previous studies have shown that hypertriglyceridemia and low HDL-C levels are more prevalent in T2DM patients than in the normal population. In contrast, high LDL-C levels were not significantly different between the two groups (17). It has also been shown that high TG levels can cause overload of free fatty acids or lipotoxicity in some organs, and lead to β-cell dysfunction and insulin resistance. At the same time, high TG levels can directly promote inflammatory response or ER stress (18). However, dyslipidemia may also be a direct cause of IR in the absence of lipotoxicity, such as inflammation, ER stress, or other mechanisms (5). In addition, low HDL-C levels may affect glucose homeostasis through direct glucose uptake, reducing insulin sensitivity and insulin secretion (19). Second, low TG/HDL-C levels were associated with an increased risk of T2DM. In fact, there is much evidence that very low TG levels or very high HDL-C levels are associated with adverse effects on health and disease. Recently, Zhong et al. pooled 37 prospective cohort studies to conclude that HDL-C levels are associated with all-cause mortality, cardiovascular disease, and cancer in a J-shaped dose-response manner in the general population, implying that extremely high HDL-C levels are associated with an increased risk of death (20). Moreover, a Danish study found a U-shaped relationship between HDL-C concentration and all-cause mortality, in which mortality was higher in individuals with very high HDL-C levels (21). It has also been found that very high HDL-C levels do not represent a good prognosis, especially in young people (22). The mechanisms by which extremely high HDL-C levels are associated with an increased risk of death remain unclear. One possible explanation is that very high HDL-C levels may be due to genetic variants, leading to adverse health effects (23–25). Another explanation is that the conformation and function of lipoproteins in people with very high HDL-C levels may be impaired, resulting in the dysfunction of high-density lipoproteins, causing harm to the human body (26). Third, individuals with normal glycemic levels tend to ignore the risk of developing T2DM. The U-shaped relationship between TG/HDL-C ratio and the incidence of T2DM suggests that inappropriate TG/HDL-C ratio may be a potential intervention target to prevent the development of impaired glucose tolerance. Therefore, TG/HDL-C levels that are either too high or too low may be harmful.

### 4.2 Strengths and limitations of this study

Our study had several advantages over other studies: (1) our study was based on the NAGALA database, which has a complete and reliable clinical dataset. The sample size for assessing the association between TG/HDL-C ratio and the risk of incident T2DM was larger, the follow-up time was longer, and the TG/HDL-C ratio in our study had a more extensive range. These preconditions allowed us to assess the association more accurately between TG/HDL-C ratio and the risk of incident T2DM; (2) the populations were different. The data in our study were based on Japanese individuals, and we excluded those with prediabetes at baseline, i.e., those with HbA1c greater than 5.7%, and those with impaired fasting glucose levels. Therefore, our study is significant for the early detection and prevention of T2DM in Japanese men with normal blood glucose levels. (3) Our study adjusted for more confounding factors to make the conclusion more reliable and accurate.

Although our study has these advantages, it has many limitations. (1) The participants of our study were Japanese men. Therefore, our results should be interpreted cautiously due to sex and racial limitations. (2) We could not adjust for variables not included in the database itself, such as low-density lipoprotein cholesterol (LDL-C) and plasma insulin, because the original data were obtained from a public database. If possible, we will collect these data to further explore their relationship with T2DM risk. (3) We defined T2DM with baseline HbA1c and FPG levels, but without an oral glucose tolerance test (OGTT), so we may have underestimated the incidence of T2DM. (4) the pathogenesis and effect of TG/HDL-C on the risk of T2DM need to be further studied. (5) The AUC of ROC curves for TG, HDL-C, and TG/HDL-C were all lower than 0.7, indicating a poor prediction effect, which may be related to the small sample size. ROC curve evaluation is intended to provide research directions for subsequent researchers, and more studies are needed to obtain a more accurate prediction effect.

## 5 Conclusions

Our study is the first to show a U-shaped relationship between baseline TG/HDL-C ratio and new-onset T2DM in Japanese men with normal glucose levels. This result has a reference value for future mechanism and clinical research in related fields.

## Data availability statement

Publicly available datasets were analyzed in this study. This data can be found here: https://datadryad.org/stash/dataset/doi:10.5061%2Fdryad.8q0p192.

## Ethics statement

The studies involving humans were approved by Murakami Memorial Hospital Ethics Committee. The studies were conducted in accordance with the local legislation and institutional requirements. The participants provided their written informed consent to participate in this study.

## Author contributions

BS contributed to the design of the study and writing most of the first draft. KW and XZ organized the database and responded to the editor and reviewers. TY, WL, and TL performed the statistical analysis. GG and HF participated in the critical modification of important knowledge content. CL initiated the study design and ensured the accuracy or completeness of all questions in the study. All authors contributed to the article and approved the submitted version.
