# Supplementary material for: A U-shaped association between the triglyceride to high-density lipoprotein cholesterol ratio and the risk of incident type 2 diabetes mellitus in Japanese men with normal glycemic levels: a population-based longitudinal cohort study
Source: Front Endocrinol (Lausanne). 2023 Sep 21;14:1180910. doi: 10.3389/fendo.2023.1180910 (PMC10552638; doi:10.3389/fendo.2023.1180910)
Supplement: Supplementary file 1 [file DataSheet_1.pdf]

Supplementary Material

1 Supplementary figures

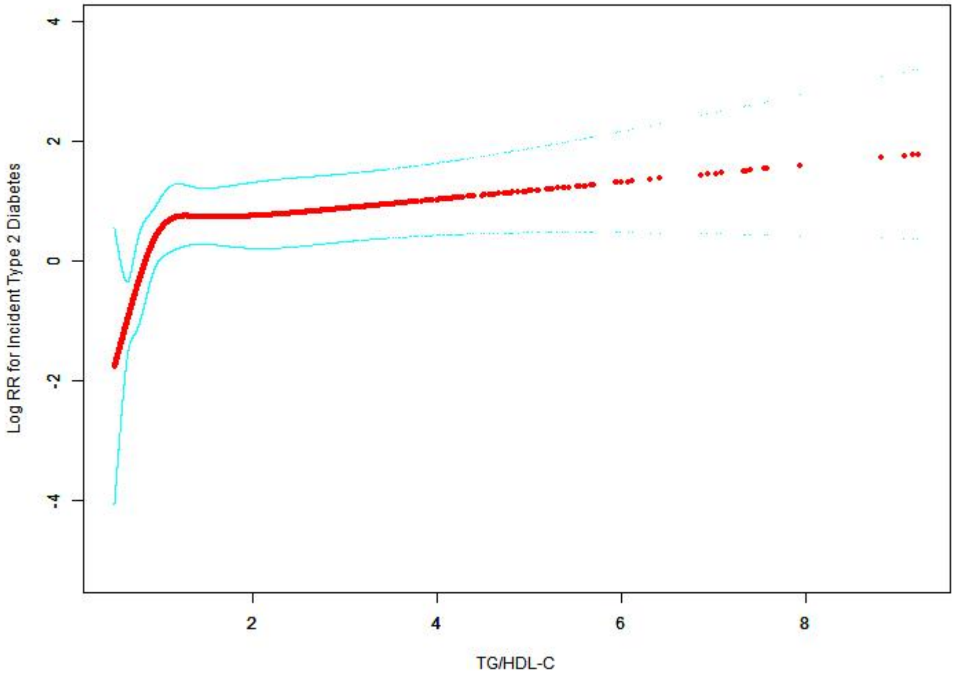

Supplementary figure 1

Association between TG/HDL-C and incident T2DM among women.

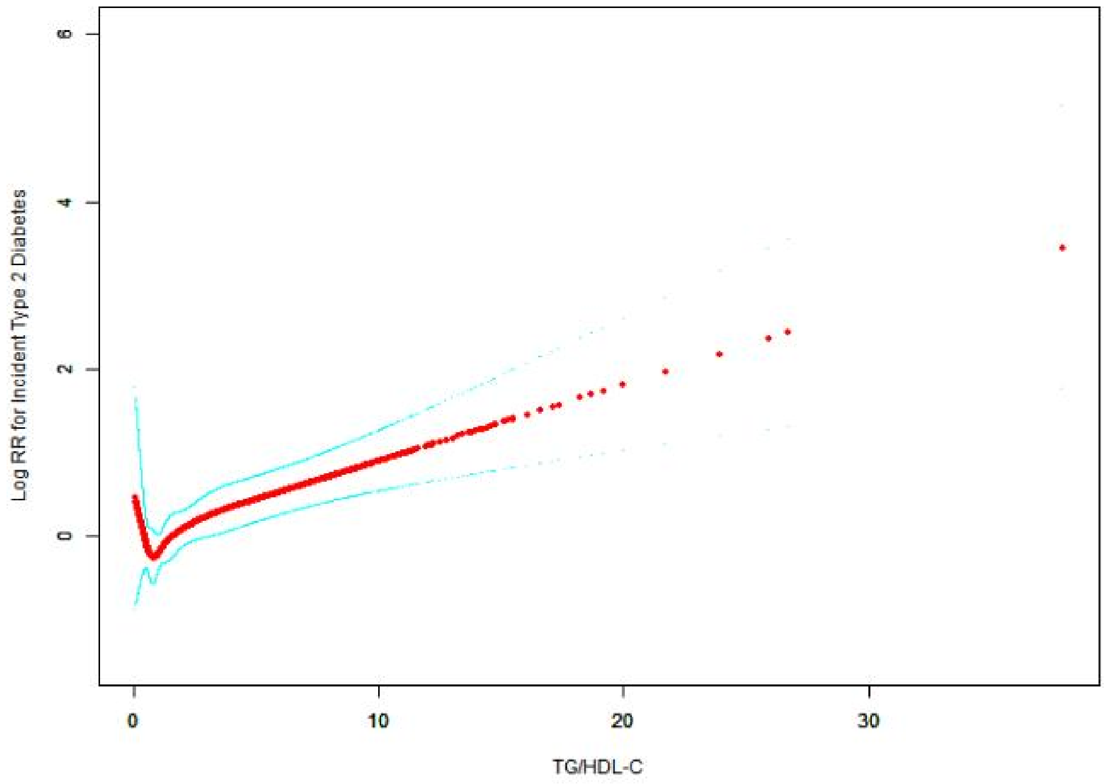

Supplementary figure 2

Association between TG/HDL-C and incident T2DM among both men and women.

2 Supplementary Tables

Supplementary table 1

	Statistics	Incident Type 2 Diabetes	***p value***
Age, yr	43.796 ± 8.902	1.043 (1.024, 1.062)	<0.00001
Fatty liver			
No	5839 (75.989%)	1	
Yes	1845 (24.011%)	4.482 (3.278, 6.129)	<0.00001

BMI, kg/m2	22.890 ± 2.911	1.223 (1.181, 1.268)	<0.00001
Waist circumference, cm	80.068 ± 7.798	1.088 (1.071, 1.105)	<0.00001
ALT, IU/L	23.329 ± 13.335	1.023 (1.017, 1.030)	<0.00001
AST, IU/L	19.423 ± 7.593	1.030 (1.018, 1.042)	<0.00001
Body Weight, kg	66.903 ± 9.738	1.054 (1.042, 1.067)	<0.00001
Fasting plasma glucose, mmol/L	5.283 ± 0.363	19.874 (12.053, 32.770)	<0.00001
Systolic blood pressure, mmHg	118.413 ± 14.005	1.030 (1.020, 1.040)	<0.00001
Diastolic blood pressure, mmHg	74.580 ± 9.882	1.044 (1.029, 1.059)	<0.00001
GGT, IU/L	25.034 ± 20.339	1.010 (1.007, 1.013)	<0.00001
HDL-cholesterol, mmol/L	1.326 ± 0.342	0.179 (0.098, 0.327)	<0.00001
Total Cholesterol, mmol/L	5.128 ± 0.843	1.255 (1.053, 1.497)	0.01135
Triglycerides, mmol/L	1.024 ± 0.548	2.327 (1.856, 2.918)	<0.00001
TG/HDL-C	1.972 ± 1.319	1.469 (1.340, 1.610)	<0.00001
HbA1c, mmol/mol	32.474 ± 3.083	1.395 (1.317, 1.478)	<0.00001
Alcohol consumption			
Never	4847 (63.079%)	1	
Light	1259 (16.385%)	0.739 (0.466, 1.173)	0.19941
Moderate	1080 (14.055%)	0.791 (0.485, 1.291)	0.34804
Severe	498 (6.481%)	1.686 (1.033, 2.752)	0.03662
Smoking status			
Never	2695 (35.073%)	1	
Past	2280 (29.672%)	0.936 (0.608, 1.440)	0.76401

Current	2709 (35.255%)	1.542 (1.074, 2.215)	0.01905
Habit of exercise 0/1			
No	6196 (80.635%)	1	
Yes	1488 (19.365%)	0.766 (0.496, 1.181)	0.22734

Continuous variables are presented as mean ± S.D. or as median (Q1-Q4). Categorical data are presented as frequencies (percentages).

Abbreviation: Q, quartile; ALT, alanine aminotransferase; AST, aspartate aminotransferase; GGT, gamma-glutamyl transpeptidase; DBP, diastolic blood pressure; SBP, systolic blood pressure; TG, triglyceride; HDL-cholesterol, high-density lipoprotein-cholesterol; TG/HDL-C, triglyceride to high-density lipoprotein cholesterol ratio; HbA1c, Hemoglobin A1c; BMI, body mass index.
